# The C-terminal domain of zDHHC2 contains distinct sorting signals that regulate intracellular localisation in neurons and neuroendocrine cells

**DOI:** 10.1016/j.mcn.2017.07.007

**Published:** 2017-12

**Authors:** Christine Salaun, Louise Ritchie, Jennifer Greaves, Trevor J. Bushell, Luke H. Chamberlain

**Affiliations:** Strathclyde Institute of Pharmacy and Biomedical Sciences, University of Strathclyde, Glasgow G4 0RE, United Kingdom

**Keywords:** PSD, post synaptic density, PSD95, post synaptic density protein 95, AKAP79/150, A kinase anchoring protein 79/150, GABAR, gamma-aminobutyric acid A receptor, AMPAR, alpha-amino-3-hydroxy-5-methyl-4-isoxazolepropionic acid receptor, NMDAR, *N*-methyl-d-aspartic acid receptor, GRIP1b, glutamate receptor interacting protein 1b, TTX, Tetrodotoxin, SNARE, soluble *N*-ethylmaleimide sensitive factor attachment protein receptor, SNAP25, Synaptosome associated protein 25, AP2, adaptor protein 2, cLTP, chemically induced long-term potentiation, TfR, transferrin receptor, Intracellular trafficking, Membrane recycling, Membrane trafficking, Post translational modification, Protein palmitoylation, zDHHC enzymes

## Abstract

The S-acyltransferase zDHHC2 mediates dynamic S-acylation of PSD95 and AKAP79/150, which impacts synaptic targeting of AMPA receptors. zDHHC2 is responsive to synaptic activity and catalyses the increased S-acylation of PSD95 that occurs following action potential blockade or application of ionotropic glutamate receptor antagonists. These treatments have been proposed to increase plasma membrane delivery of zDHHC2 via an endosomal cycling pathway, enhancing substrate accessibility. To generate an improved understanding of zDHHC2 trafficking and how this might be regulated by neuronal activity, we searched for intramolecular signals that regulate enzyme localisation. Two signals were mapped to the C-terminal tail of zDHHC2: a non-canonical dileucine motif [SxxxLL] and a downstream NP motif. Mutation of these signals enhanced plasma membrane accumulation of zDHHC2 in both neuroendocrine PC12 cells and rat hippocampal neurons, consistent with reduced endocytic retrieval. Furthermore, mutation of these signals also increased accumulation of the enzyme in neurites. Interestingly, several threonine and serine residues are adjacent to these sorting motifs and analysis of phospho-mimetic mutants highlighted a potential role for phosphorylation in regulating the efficacy of these signals. This study offers new molecular insight into the signals that determine zDHHC2 localisation and highlights a potential mechanism to regulate these trafficking signals.

## Highlights

• Dynamic trafficking of zDHHC2 regulates the localisation of this S-acylation enzyme and controls access to its substrates.
• Two separate (and atypical) sequences were identified within the C-terminal tail of zDHHC2 that affect enzyme localisation.
• Mutating these motifs induced the accumulation of zDHHC2 at the plasma membrane of hippocampal neurons and PC12 cells.
• Phosphorylation may be a potential mechanism to regulate the efficacy of these sorting signals.

## 1 Introduction

S-acylation (or *palmitoylation*) is a reversible post-translational modification involving the attachment of fatty acids onto cysteine residues (Salaun et al., 2010), which regulates the stability, localisation and function of a broad array of different proteins (Salaun et al., 2010, Linder and Deschenes, 2007, Chamberlain and Shipston, 2015). The “zDHHC” (zinc finger aspartate-histidine-histidine-cysteine) family of enzymes mediate essentially all S-acylation reactions that occur in cells (Fukata et al., 2004, Roth et al., 2006). Although we lack a comprehensive understanding about which of the twenty-four zDHHC enzymes modify the several hundred S-acylated proteins in mammalian cells, many enzyme-substrate pairs have been characterized (Greaves and Chamberlain, 2011a).

Recent studies have uncovered an intriguing role for S-acylation in the regulation of neuronal physiology (Fukata and Fukata, 2010). In particular, this modification has been suggested to play an important role in the regulation of neurotransmitter receptors, including the γ-subunit of GABA(A) receptors (Keller et al., 2004), and AMPA and NMDA glutamate receptor (AMPAR and NMDAR) subunits (Hayashi et al., 2005, Hayashi et al., 2009).

AMPARs are regulated directly by S-acylation at two sites, which controls receptor localisation at synapses and trafficking of newly-synthesized protein through the secretory pathway in the cell soma (Hayashi et al., 2005). In addition to direct modification of AMPAR subunits, the synaptic targeting of these receptors is also thought to be regulated by the S-acylation of a number of other accessory proteins, including GRIP1b (Thomas et al., 2012), AKAP79/150 (Woolfrey et al., 2015) and PSD95 (El-Husseini, 2002). S-acylation of GRIP1b and AKAP79/150 targets these proteins to recycling endosomes, where they regulate AMPAR recycling and exocytosis (Thomas et al., 2012, Woolfrey et al., 2015). S-acylation of PSD95 is important for targeting of this molecular scaffold to post-synaptic regions, where it regulates synaptic clustering of AMPAR (Craven et al., 1999), indirectly via interaction with the Stargazin protein (Chen et al., 2000).

S-acylation of these proteins is mediated by a sub-set of zDHHC enzymes that are localised to dendritic regions, including zDHHC-5 and -8, which modify GRIP1b (Thomas et al., 2012), and zDHHC2, which S-acylates both AKAP79/150 and PSD95 (Woolfrey et al., 2015, Noritake et al., 2009). The S-acylation of PSD95 is regulated by neuronal activity and the block of action potential generation using tetrodotoxin (TTX) or inhibiting synaptic activity using ionotropic glutamate receptor antagonists promotes an increase in palmitate incorporation into this molecular scaffold (El-Husseini, 2002, Noritake et al., 2009). Importantly, these activity-dependent changes in PSD-95 S-acylation were shown to be prevented by knock-down of zDHHC2 (Noritake et al., 2009). The increased S-acylation of PSD95 following TTX treatment is not thought to reflect an enhanced intrinsic activity of zDHHC2 but rather a change in the intracellular localisation of this enzyme (Noritake et al., 2009): total internal reflection microscopy suggested that zDHHC2 translocates to the plasma membrane in response to reduced neuronal activity (Noritake et al., 2009). Indeed we previously showed that zDHHC2 enters a dynamic cycling pathway that traffics the enzyme between recycling endosomes and the plasma membrane in neuroendocrine PC12 cells (Greaves et al., 2011), and we proposed that altered flux of zDHHC2 through this pathway is likely to underlie activity-dependent change in zDHHC2 localisation (Greaves et al., 2011). Signals that regulate zDHHC2 trafficking were suggested to reside in the C-terminus of the enzyme (Greaves et al., 2011).

There is little information available on how synaptic activity regulates protein localisation. As first step toward defining the basis of this for zDHHC2, we have sought to identify the intramolecular signals that control trafficking of this enzyme. As our previous work found that the cytosolic C-terminus of zDHHC2 was important for its intracellular targeting, we have therefore analysed this region in more details. We report the identification of two novel non-canonical endocytic signals in the C-terminus of zDHHC2 and highlight the potential for their regulation by phosphorylation.

## 2 Methods

### 2.1 Plasmid constructs

N-terminal EGFP- and mCHERRY-tagged murine zDHHC2 constructs were previously described (Greaves et al., 2011). The coding sequence of zDHHC2 was altered by using site-directed mutagenesis and protein truncations were generated by the introduction of premature stop codons into the appropriate region of the coding sequence. EGFP-tagged murine zDHHC5 was generated by subcloning the zDHHC5 cDNA (Fukata et al., 2004) in pEGFP-C2 (Clontech). Rab11-GFP was provided by Professor Giampietro Schiavo.

### 2.2 PC12 cell culture, transfection and fixation

PC12 cells were grown in RPMI1640 media containing 10% horse serum and 5% foetal calf serum in a humidified atmosphere containing 5% CO_2_. Cells were plated on BD poly-d-lysine coated coverslips and transfected using Lipofectamine 2000 (Life Technologies) with 0.2 μg of each plasmid construct for two days. The cells were then washed in PBS and fixed in 4% formaldehyde for 30 min. The cells were then washed again in PBS and mounted on glass slides in Mowiol.

### 2.3 Isolation, culture and transfection of hippocampal neurons

Two day old Sprague-Dawley rat pups were killed by cervical dislocation and decapitated. Hippocampi were extracted and digested in a solution containing 0.15% (w/v) papain (Sigma) at 37 °C for 20 min. Cells were then triturated with 3 glass Pasteur pipettes of decreasing diameters in 1% BSA (Sigma), pelleted and resuspended in Neurobasal-A medium (Invitrogen) supplemented with 2% B27 (Gibco) and 2 mM l-Glutamine (Gibco). 3 × 10^5^ cells were plated on 6 mm glass coverslips coated with poly-l-lysine (10 ng/ml, Sigma), and grown in a humidified atmosphere at 37 °C with 5% CO_2_ for 5 days in vitro (DIV). Cytosine-d-arabinofuranoside (5 μM, Sigma) was then added to the cultures at 5 DIV to inhibit glial cell proliferation. Neurons were transfected at 10 to 11 DIV with 0.4 μg of each plasmid using Lipofectamine 2000 in a medium composed of 50% fresh medium, 50% conditioned medium. After 4 h, the medium was replaced with 50% fresh medium, 50% conditioned medium. The cells were fixed and analysed 48 to 72 h post-transfection.

### 2.4 Confocal microscopy and image analysis

All microscopy analysis was performed on a Leica SP5 confocal microscope. Acquired image stacks were deconvolved using Huygen's software for presentation purposes. For PC12 cells, a single slice was chosen for representation purpose only and on the main criterion that the image chosen should clearly show the characteristic endosomal region as well as the plasma membrane. A max z-projection of a stack was chosen to illustrate neuron images. For presentation purposes, the images representing GFP and mCHERRY fluorescence were subjected to identical brightness and contrast adjustments.

All image quantification was performed with the Fiji software (Schindelin et al., 2012). Briefly, a z-projection of the SUM of the slices of a full stack was generated for each PC12 cell. Two regions of interest were drawn, one for the whole cell and one around the recycling endosomes (defined by the localisation of mCHERRY-wt zDHHC2). The intensity of fluorescence of each region was quantified for each channel (GFP_endo_ and mCHERRY_endo_, GFP_cell_ and mCHERRY_cell_). The index of endosomal localisation of the mutant GFP-tagged protein relative to the mCHERRY-tagged wt protein was then calculated as [(GFP_endo_/(GFP_cell_ − GFP_endo_)]/[(mCHERRY_endo_/(mCHERRY_cell_ − mCHERRY_endo_)], and finally normalized to the average ratio of the control GFP-tagged wt protein. The quantification of the endosomal localisation of the mutant GFP-tagged proteins expressed in neuronal cells was performed identically except that the region of interest for the whole cell was limited to the cell body of the neuron. The ‘endosomal region’ was defined as the intracellular region in which mCHERRY-zDHHC2 fluorescence was enriched. The expression of the proteins in neurites relative to the soma was performed as follows: a region of interest measuring 15 μm (measured from the basis of the neurite at the cell body) was drawn around several dendrites, the mean intensity of fluorescence was measured in each channel and averaged per dendrite. This number was then divided by the mean intensity of fluorescence of the soma. The relative expression of the GFP-tagged mutant proteins in neurites was given by the following ratio: (GFP_neurite_/GFP_soma_)/(mCHERRY_neurite_/(mCHERRY_soma_), and then normalized with the ratio calculated for the GFP-tagged wt protein. At least 2 experiments were performed for each construct. Statistical analyses were generated with GraphPad Prism using ANOVA analyses followed by a Dunnett's post hoc test or Student's *t*-test as appropriate (not significant, p > 0.05; ^⁎^, p ≤ 0.05; ^⁎⁎^, p ≤ 0.01; ^⁎⁎⁎^, p ≤ 0.001).

## 3 Results

Dynamic changes in zDHHC2 localisation are thought to be central to activity-dependent S-acylation changes occurring in neuronal dendrites (Noritake et al., 2009, Fukata et al., 2013, Greaves et al., 2011). Our previous study showed that zDHHC2 cycles between the plasma membrane and recycling endosomes and that sorting signals regulating this dynamic localisation are located in the C-terminal tail of the enzyme (Greaves et al., 2011). To investigate the nature of the signals that regulate zDHHC2 distribution between recycling endosomes and the plasma membrane, we employed a quantitative approach that we previously used to identify changes in the endosome/plasma membrane distribution of the SNARE protein SNAP25 (Greaves and Chamberlain, 2011b). This method involves co-expressing wild-type mCHERRY-tagged proteins together with mutant GFP-tagged proteins and comparing their endosomal fluorescence intensity relative to whole cell fluorescence.

**Fig. 1:**
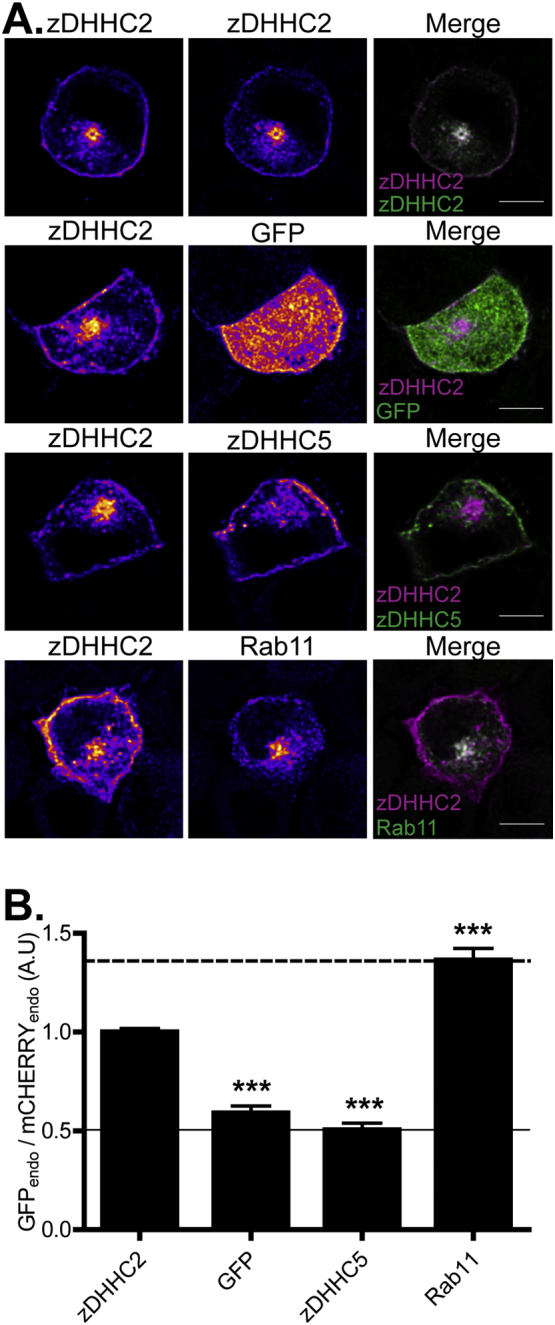
Intracellular localisation of wt zDHHC2 in PC12 cells and comparison with various control intracellular markers. (A) PC12 cells were co-transfected with a mCHERRY-zDHHC2 plasmid and various constructs encoding GFP-tagged proteins. GFP is used as a marker for the cytosol, zDHHC5 for the plasma membrane and Rab11 for recycling endosomes. The *left* panel shows the localisation of the wt mCHERRY-tagged zDHHC2 and the *middle* panel the expression of the co-expressed GFP-tagged proteins. Both are represented in FIRE pseudocolour. The right panel is the merge of the left and middle panels, with the wt mCHERRY-zDHHC2 presented in Magenta and GFP-tagged proteins in Green. Scale bar represents 5 μm. (B) The graph shows the normalized mean ratio ± SEM of endosomal fluorescence of the GFP constructs relative to mCHERRY-zDHHC2 (n = 15 zDHHC2 cells, 16 GFP cells, 17 zDHHC5 cells and 20 Rab11 cells). Statistical analysis (ANOVA) shows a significant difference in the localisation of the various markers compared to zDHHC2 (^⁎⁎⁎^, p ≤ 0.001). The lines represent the upper and lower ratio of endosomal enrichment that can be calculated (dashed line, Rab 11 vs zDHHC2, value of 1.36, filled line, zDHHC5 vs zDHHC2, value of 0.576). These maximum and minimum levels have been added to every subsequent graph in order to provide easier comparison between different figures. (For interpretation of the references to colour in this figure legend, the reader is referred to the web version of this article.)

A value for [ENDO fluorescence/(total cell fluorescence − ENDO fluorescence)] was calculated for each fluorophore (“GFP_endo_” and “mCHERRY_endo_”), which is then expressed as a ratio (GFP_endo_/mCHERRY_endo_). Using this approach, when GFP-tagged mutant proteins have an increased endosomal fluorescence relative to mCHERRY-wild-type protein, there is an increase in the calculated ratio and vice versa. Using normalized data obtained from transfected PC12 cells, Fig. 1 shows the enrichment of Rab11 and the de-enrichment of GFP and zDHHC5 on recycling endosomes relative to zDHHC2.

### 3.1 Truncations at the C-terminus of zDHHC2 enhance plasma membrane localisation

**Fig. 2:**
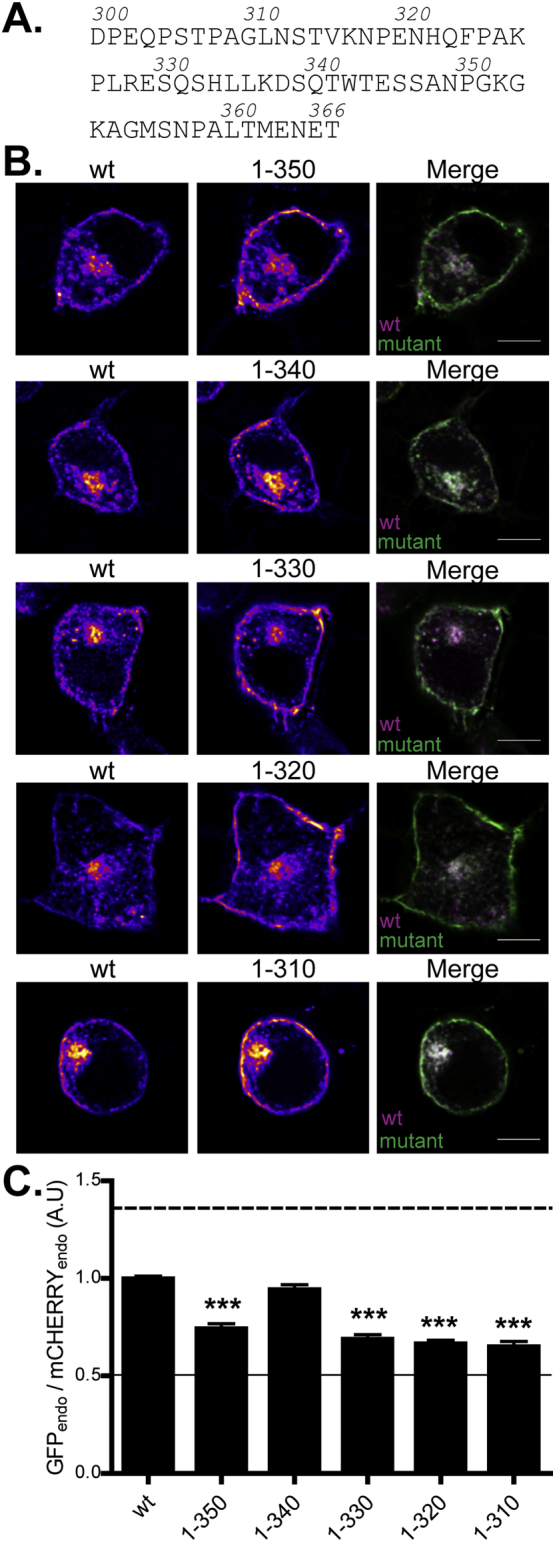
Localisation of zDHHC2 truncation constructs. (A) Amino acid sequence of the C-terminal cytosolic tail of mouse zDHHC2. The numbers indicate the position of the amino acid upstream of the inserted STOP codon, allowing for the expression of zDHHC2 truncated proteins. (B) Representative confocal images of PC12 cells co-expressing the wt mCHERRY-zDHHC2 protein (*left panel*) and the GFP-tagged truncation mutants of zDHHC2 (*middle panel*). Both are represented in FIRE pseudocolour. The right panel is the merge of the left and middle panels, with the wt mCHERRY-zDHHC2 presented in Magenta and GFP-tagged proteins in Green. Scale bar represents 5 μm. (C) Quantification of the endosomal depletion of the truncation mutants. The fluorescence intensity value of the recycling endosome compartment (endo) as a fraction of the whole cell fluorescence was calculated as described in material and methods for GFP constructs relative to the wt zDHHC2 mCHERRY-tagged protein (n = 14 wt zDHHC2 cells, 16 (1–350) cells, 15 (1–340) cells, 14 (1–330) cells, 15 (1–320) cells and 17 (1–310) cells). Statistical analysis (ANOVA) shows a significant difference in the localisation of several truncation mutants compared to wt zDHHC2 (^⁎⁎⁎^, p ≤ 0.001). The dashed and filled lines represent the upper and lower ratios of endosomal enrichment that were calculated in Fig. 1. (For interpretation of the references to colour in this figure legend, the reader is referred to the web version of this article.)

To investigate the nature of the signals in zDHHC2 that regulate intracellular targeting, we generated a number of truncations in the C-terminus of zDHHC2, as this region of the protein has previously been identified as important for enzyme localisation (Greaves et al., 2011). Quantitative analysis showed that removal of the terminal 17 amino acids (1–350 mutant) enhanced the plasma membrane localisation of the enzyme compared with wild-type zDHHC2 (Fig. 2), suggesting that this region of the enzyme might contain an endocytic sorting signal. Furthermore, comparison of the intracellular localisation of mutants 1–330 and 1–340 indicated that an additional sorting signal might be located in the amino acid region 330–340 (Fig. 2).

### 3.2 Site-directed mutagenesis identifies two sorting signals in the zDHHC2 C-terminal tail

**Fig. 3:**
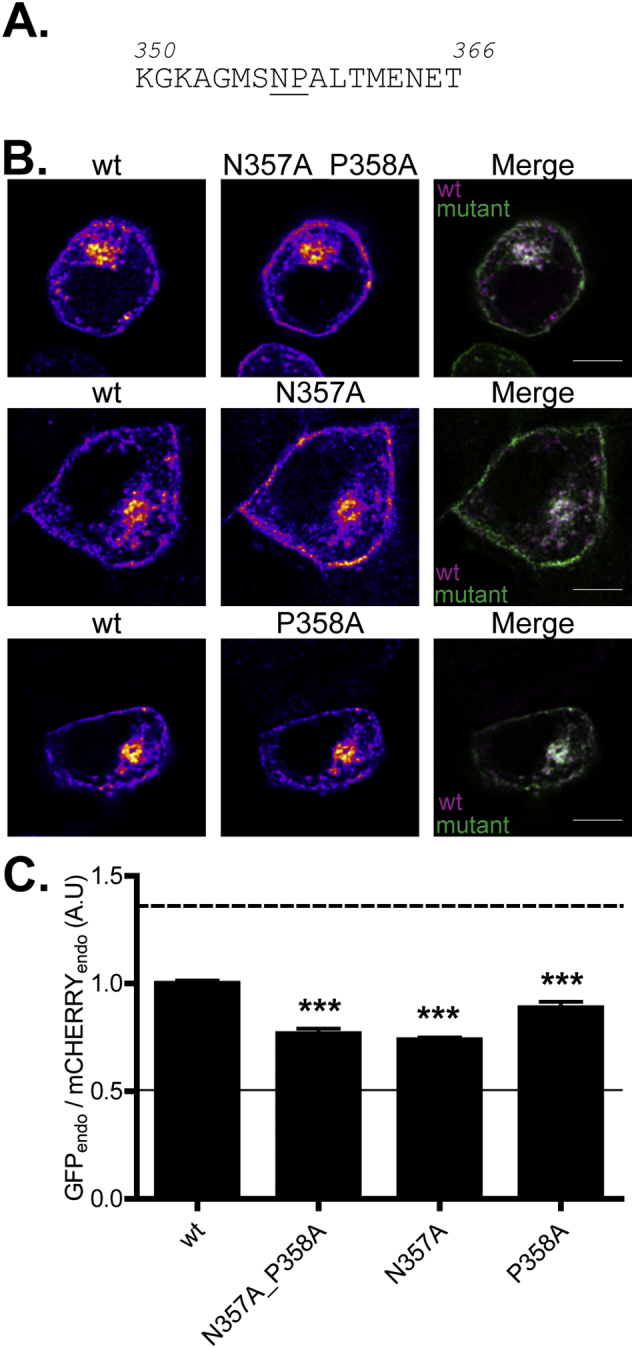
Identification of an endocytic NP motif in the C-terminal tail of zDHHC2. (A) Amino acid sequence of the 350–366 region of zDHHC2; the NP motif is underlined. (B) Representative confocal images of PC12 cells co-expressing wt mCHERRY-zDHHC2 (*left panel*) and various GFP-tagged mutants (*middle panel*). Both are represented in FIRE pseudocolour. The right panel is the merge of the left and middle panels, with the wt mCHERRY-zDHHC2 presented in Magenta and GFP-tagged proteins in Green. Scale bar represents 5 μm. (C) Quantification of the endosomal depletion of the mutants. The fluorescence intensity values of the recycling endosome compartment (endo) as a fraction of the whole cell fluorescence were calculated as described in material and methods for both GFP and mCHERRY. The graph shows the normalized mean ratio ± SEM of endosomal fluorescence of the GFP construct relative to the wt zDHHC2 mCHERRY tagged protein (n = 20 wt zDHHC2 cells, 11 N356A_P358A cells, 11 N357A cells and 10 P358A cells). Statistical analysis (ANOVA) shows a significant difference in the localisation of the mutants compared to wt zDHHC2 (^⁎⁎^, p ≤ 0.01; ^⁎⁎⁎^, p ≤ 0.001). The dashed and filled lines represent the upper and lower ratios of endosomal enrichment that were calculated in Fig. 1. (For interpretation of the references to colour in this figure legend, the reader is referred to the web version of this article.)

Analysis of the amino acid sequence downstream of residue 350 in zDHHC2 highlighted a N^357^P^358^AL motif resembling the known NPxY internalisation signal. Thus, we mutated the conserved NP amino acids to alanine (N357A_P358A) and found that this mutant was enriched at the plasma membrane relative to wild-type zDHHC2 (Fig. 3). A single N357A mutation had the same level of effect as the N357A_P358A double mutation; a single P358A mutation also significantly enhanced plasma membrane staining, albeit to a smaller level than the N357A mutation (Fig. 3).

**Fig. 4:**
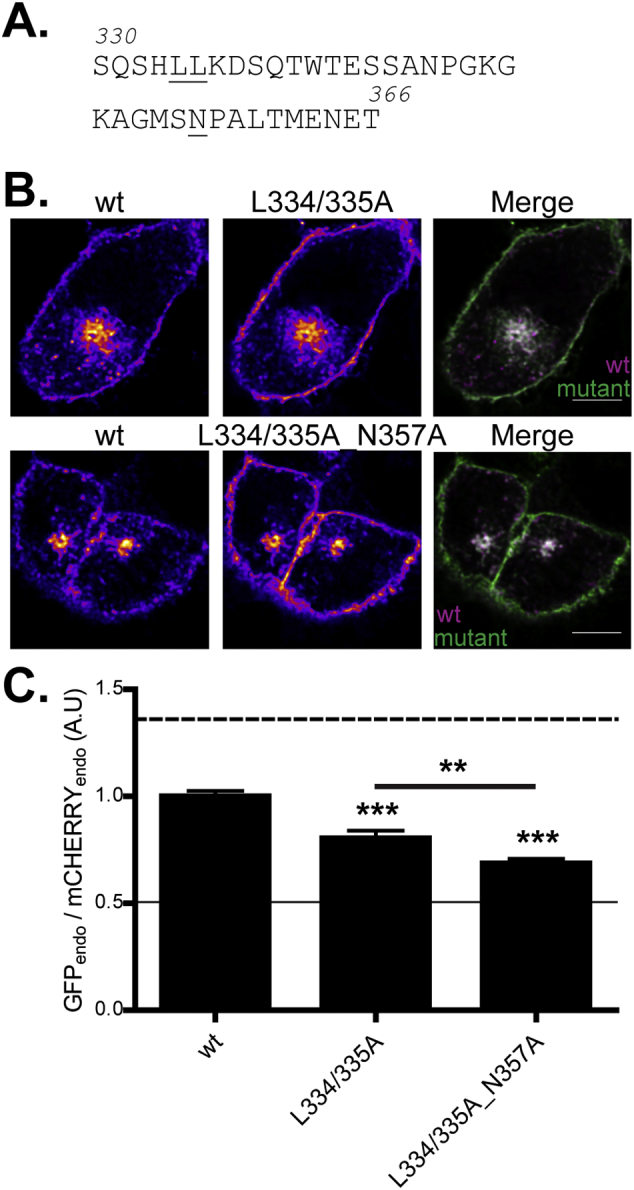
Identification of an endocytic LL motif in the C-terminal tail of zDHHC2. (A) Amino acid sequence of the 330–366 region of zDHHC2; the mutated residues are underlined. (B) Representative confocal images of PC12 cells co-expressing the wt mCHERRY-zDHHC2 protein (*left panel*) and the various GFP-tagged mutants of zDHHC2 (*middle panel*). Both are represented in FIRE pseudocolour. The right panel is the merge of the left and middle panels, with the wt mCHERRY-zDHHC2 coloured in Magenta and GFP-tagged proteins coloured in Green. Scale bar represents 5 μm. (C) Quantification of the endosomal depletion of the mutants. The fluorescence intensity values of the recycling endosome compartment (endo) as a fraction of the whole cell fluorescence were calculated as described in material and methods for both GFP and mCHERRY. The graph shows the normalized mean ratio ± SEM of endosomal fluorescence of the GFP construct relative to the wt zDHHC2 mCHERRY-tagged protein (n = 11 wt zDHHC2 cells, 11 L334/335A cells and 13 L334/335A_N357A cells). Statistical analysis (ANOVA) shows a significant difference in the localisation of the mutants compared to wt zDHHC2 (^⁎⁎⁎^, p ≤ 0.001), as well as between the L334/335A mutant and the L334/335A_N357A mutant (^⁎⁎^, p ≤ 0.01). The dashed and filled lines represent the upper and lower ratios of endosomal enrichment/depletion that were calculated in Fig. 1. (For interpretation of the references to colour in this figure legend, the reader is referred to the web version of this article.)

Results from truncation analysis also suggest the presence of an additional sorting motif in zDHHC2 between amino acids 331–340. Interestingly a dileucine motif (L334_L335) is present in this region of zDHHC2 although this motif does not conform to the consensus for a dileucine endocytic signal (D/ExxxLL). Nevertheless, mutation of these two leucine residues to alanine (L334/335A) led to an increased association of zDHHC2 with the plasma membrane (Fig. 4). Furthermore, the combined mutation of the NP and LL signals (L334/335A_N357A) led to an even greater enrichment at the plasma membrane, showing that these signals are independent of each other (Fig. 4).

### 3.3 The LL and NP motifs regulate zDHHC2 localisation in hippocampal neurons

**Fig. 5:**
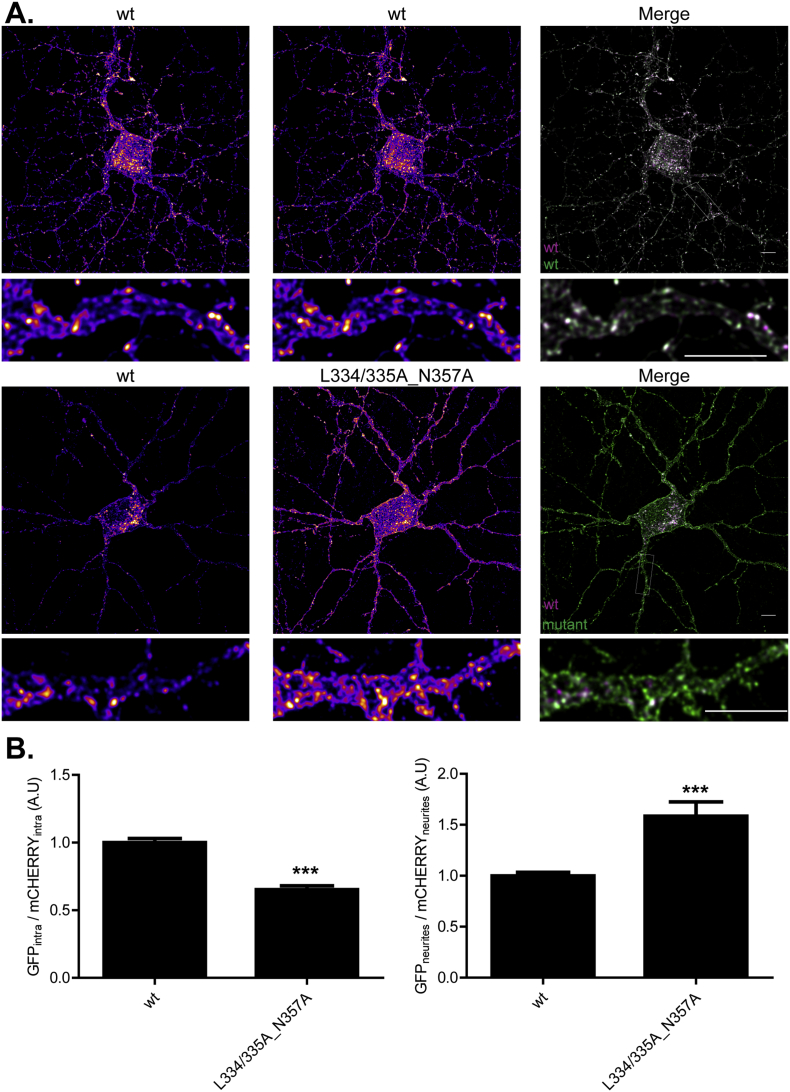
The mutation of the LL and NP sites affects the localisation of zDHHC2 in primary rat hippocampal neurons. (A) Representative confocal images of 13 DIV primary rat hippocampal neurons cells co-expressing wt mCHERRY-zDHHC2 (*left panel*) and the GFP tagged wt or endocytic mutant of zDHHC2 (*middle panel*). Both are represented in FIRE pseudocolour. Below each cell is a zoom image of a neurite region (defined by a box in the corresponding full size merged image). The right panel is the merge of the left and middle panels, with the wt mCHERRY-zDHHC2 coloured in Magenta and GFP tagged proteins coloured in Green. Scale bar represents 5 μm. (B) Quantification of the depletion of the mutant of zDHHC2 from intracellular membranes and of its neurite enrichment. *Left panel*. The fluorescence intensity values of the intracellular compartments (intra) as a fraction of the whole soma fluorescence were calculated as described in material and methods for both GFP and mCHERRY. The graph shows the normalized mean ratio ± SEM of intracellular fluorescence of the GFP construct relative to the wt zDHHC2 mCHERRY-tagged protein. *Right panel*. The mean fluorescence intensity of the neurites relative to the soma was calculated for both GFP- and mCHERRY-tagged proteins. The graph shows the normalized ratio ± SEM of neurite fluorescence intensity for the GFP construct relative to the wt mCHERRY zDHHC2 protein (n = 13 wt zDHHC2 cells and 14 L334/335A_N357A cells). Statistical analysis (Student's *t-*test) shows a significant difference in the localisation of the mutant compared to wt zDHHC2 (^⁎⁎⁎^, p ≤ 0.001). (For interpretation of the references to colour in this figure legend, the reader is referred to the web version of this article.)

To investigate if the dileucine and NP motifs also function as sorting motifs in neurons, we expressed mCHERRY-tagged wild-type zDHHC2 together with the GFP-tagged L334/335A_N357A mutant in primary rat hippocampal neurons. Within neurons, the localisation of GFP- and mCHERRY-tagged wild-type zDHHC2 was broadly similar. However there was a marked enrichment of the L334/335A_N357A mutant at the plasma membrane, which was evident in both the cell body and neurites (Fig. 5). Furthermore, we observed that the L334/335A_N357A mutant appeared to exhibit an increased expression in neurites compared to the soma. To assess this quantitatively, we expressed the mean fluorescence intensity of the neurites relative to the fluorescence intensity of the cell body, which revealed a significant relative increase in the level of the L334/335A_N357A mutant in neurites (Fig. 5).

### 3.4 Mutation of serine and threonine residues in proximity to the LL and NP motifs affects zDHHC2 localisation in PC12 cells

**Fig. 6:**
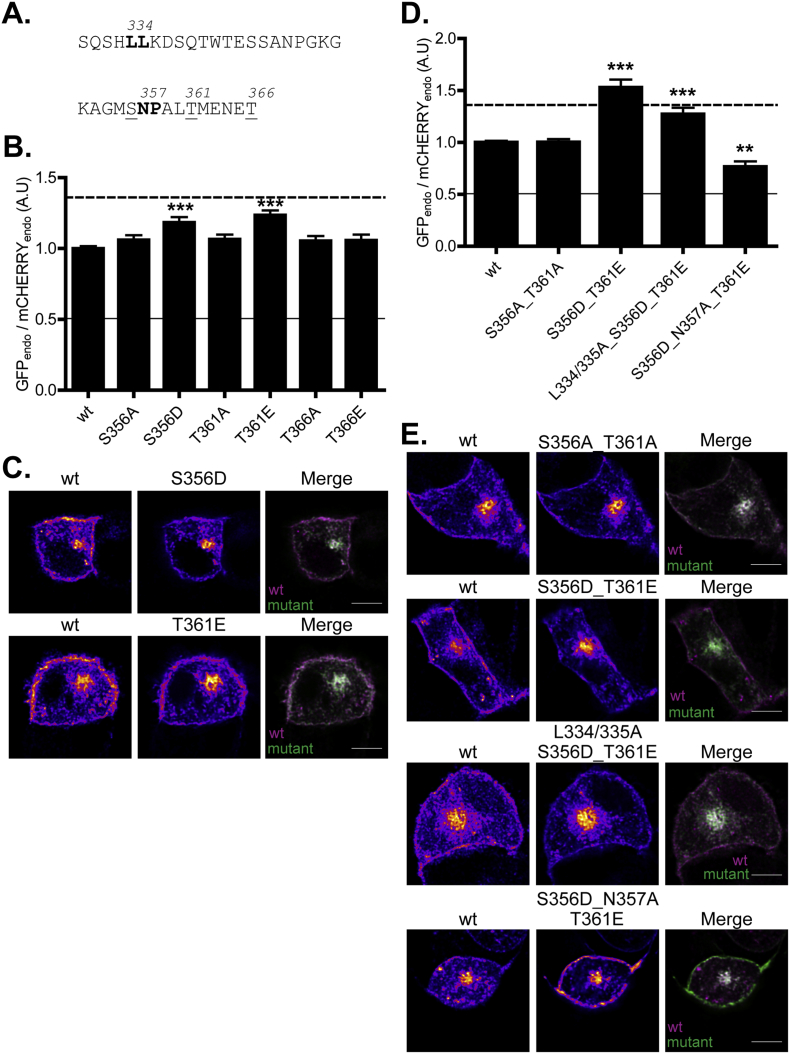
Phospho-mimetic mutations of serine and threonine residues surrounding the N357_P358 endocytic site enrich zDHHC2 in endosomes. (A) Amino acid sequence of the 330–366 region of zDHHC2. The endocytic motifs are highlighted in bold and the serine and threonine residues that were mutated are underlined. (B) Quantification of the endosomal enrichment of the mutants. The fluorescence intensity values of the recycling endosome compartment (endo) as a fraction of the whole cell fluorescence were calculated as described in material and methods for both GFP and mCHERRY. The graph shows the normalized mean ratio ± SEM of endosomal fluorescence of the GFP constructs relative to wt mCHERRY-zDHHC2 (n = 16 wt zDHHC2 cells, 10 S356A cells, 12 S356D cells, 10 T361A cells, 12 T361E cells, 13 T366A cells and 11 T366D cells). Statistical analysis (ANOVA) shows a significant difference in the localisation of some mutants compared to wt zDHHC2 (^⁎⁎⁎^, p ≤ 0.001). The dashed and filled lines represent the upper and lower ratios of endosomal enrichment/depletion that were calculated in Fig. 1. (C) Representative confocal images of PC12 cells co-expressing the wt mCHERRY-zDHHC2 protein (*left panel*) and the two GFP-tagged mutants that were significantly differently localised than the wt protein (*middle panel*). Both are represented in FIRE pseudocolour. The right panel is the merge of the left and middle panels, with the wt mCHERRY-zDHHC2 coloured in Magenta and GFP-tagged proteins coloured in Green. Scale bar represents 5 μm. (D) Evidence that the phospho-mimetic mutations affect the activity of the N357_P358 endocytic motif but do not affect the activity of the L334/335 site. The fluorescence intensity values were processed, analysed and presented as described in (B) (n = 15 wt zDHHC2 cells, 11 S356A_T361A cells, 11 S356D_T361E cells, 10 L334/335A_S356D_T361E cells and 11 S356D_T361E_N357A cells). (E) Representative confocal images of PC12 cells are presented as described in (C). (For interpretation of the references to colour in this figure legend, the reader is referred to the web version of this article.)

As the localisation of zDHHC2 in hippocampal neurons is modulated by synaptic activity, we investigated if the identified endocytic signals might be subject to regulation. A number of phosphorylation sites have been identified in zDHHC2 in phosphoproteomic studies, including serine-356 (S357 in human zDHHC2, http://www.phosphosite.org/proteinAction?id=24126&showAllSites=true), which is immediately upstream of the NP motif (Fig. 6). In addition there are two threonine residues three amino acids downstream from the NP motif (Thr-361 and Thr-366), although there is currently no evidence that these residues are subject to phosphorylation. We investigated how phospho-mimetic and phospho-null mutations of these three residues affect zDHHC2 localisation. Interestingly, S356D and T361E phospho-mimetic mutations both led to a loss of plasma membrane staining of zDHHC2, consistent with an increased rate of endocytosis, whereas phospho-null mutations (S356A and T361A) had no effect (Fig. 6). The loss of plasma membrane localisation of the S356D_T361E mutant was only observed in the presence of an intact NP motif: when these mutations were combined with a N357A mutation, zDHHC2 accumulated at the plasma membrane to a similar extent as the N357A single mutant (Fig. 6). In contrast, combining the S356D_T361E mutations with leucine-to-alanine substitutions in the identified dileucine endocytic signal had an additive effect (Fig. 6). These results suggest that the effects of phospho-mimetic mutations at S356 and T361 are via modulation of the efficacy of the NP endocytic signal.

**Fig. 7:**
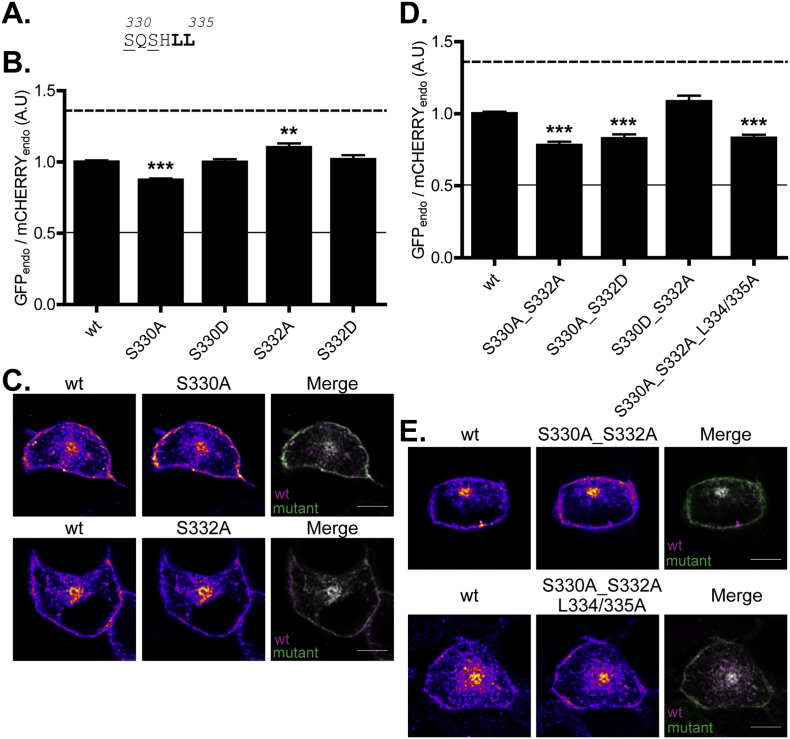
A phospho-mimetic mutation of S330 converts the SxxxLL motif into a classic D/ExxxLL motif whereas a phospho-null mutation depletes zDHHC2 from endosomes. (A) Amino acid sequence of the 330–335 region of zDHHC2. The endocytic L334/335 motif is in bold and the serine residues that were mutated are underlined. (B) Quantification of the endosomal depletion or enrichment of the mutants. The fluorescence intensity values of the recycling endosome compartment (endo) as a fraction of the whole cell fluorescence were calculated as described in material and methods for both GFP and mCHERRY. The graph shows the normalized mean ratio ± SEM of endosomal fluorescence of the GFP construct relative to the wt zDHHC2 mCHERRY-tagged protein (n = 24 wt zDHHC2 cells, 19 S330A cells, 17 S330D cells, 17 S332A cells and 19 S332D cells). Statistical analysis (ANOVA) shows a significant difference in the localisation of some mutants compared to wt zDHHC2 (^⁎⁎^, p ≤ 0.01; ^⁎⁎⁎^, p ≤ 0.001). The dashed and filled lines represent the upper and lower ratios of endosomal enrichment/depletion that were calculated in Fig. 1. (C) Representative confocal images of PC12 cells co-expressing the wt mCHERRY-zDHHC2 protein (*left panel*) and the two GFP-tagged mutants of zDHHC2 that were found to be significantly differently localised than the wt protein (*middle panel*). Both are represented in FIRE pseudocolour. The right panel is the merge of the left and middle panels, with the wt mCHERRY-zDHHC2 coloured in Magenta and GFP-tagged proteins coloured in Green. Scale bar represents 5 μm. (D) Evidence that S330 is part of the L334/335 endocytic motif. The fluorescence intensity values were processed, analysed and presented as described in (B) (n = 17 wt zDHHC2 cells, 9 S330A_S332A cells, 10 S330A_S332D cells, 11 S330D_S332A cells and 12 S330A_S332A_L334/335A cells). (E) Representative confocal images of PC12 cells are presented as described in (C). (For interpretation of the references to colour in this figure legend, the reader is referred to the web version of this article.)

To examine if the dileucine motif of zDHHC2 might also be regulated by phosphorylation of neighbouring residues we searched for identified phosphorylation sites in this region of the protein. Interestingly, serine-332, two amino acids upstream from the leucine residues, has been identified as undergoing phosphorylation (S333 in human zDHHC2) (Fig. 7). We were also interested in the possibility that phosphorylation of serine-330, which is present four amino acids upstream from the leucines might create a motif that is more akin to the consensus dileucine signal (i.e. D/ExxxLL) by introducing a negative charge at the − 4 position. Again, we observed that the introduction of phospho-mimetic or phospho-null mutations at these residues differentially affected the localisation of zDHHC2. Indeed, whereas mutation of S330 to an alanine (S330A) led to increased accumulation of the enzyme at the plasma membrane, consistent with a loss of function of the dileucine motif, the phospho-mimetic S330D mutation had no effect on zDHHC2 localisation (Fig. 7). This effect of the S330A mutation was observed when combined with either S332A or S332D mutations (Fig. 7). Importantly, the effects of a double phospho-null mutation (S330_S332A) and mutation of the leucine residues (L334/335A) were non-additive arguing that the observed effects of serine mutations are via modulation of the efficacy of the dileucine endocytic signal (Fig. 7).

**Fig. 8:**
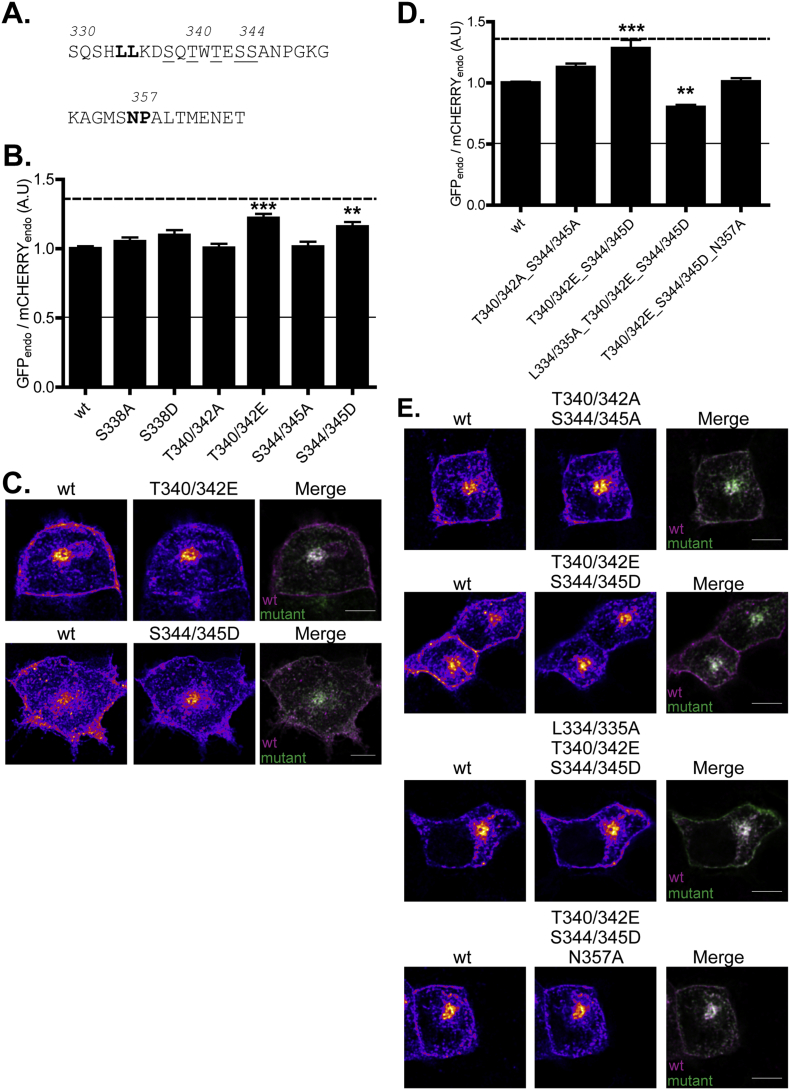
Phospho-mimetic mutations of serine and threonine residues downstream of the L334/335 endocytic site enrich zDHHC2 in endosomes. (A) Amino acid sequence of the 330–366 region of zDHHC2. The two endocytic motifs are highlighted in bold and the serine and threonine residues that were mutated are underlined. (B) Quantification of the endosomal enrichment or depletion of the mutants. The fluorescence intensity values of the recycling endosome compartment (endo) as a fraction of the whole cell fluorescence were calculated as described in material and methods for both GFP and mCHERRY. The graph shows the normalized mean ratio ± SEM of endosomal fluorescence of the GFP construct relative to the wt zDHHC2 mCHERRY-tagged protein (n = 18 wt zDHHC2 cells, 12 S338A cells, 10 S338D cells, 12 T340/342A cells, 11 T340/342E, 10 S344/345A cells and 10 S344/345D cells). Statistical analysis (ANOVA) shows a significant difference in the localisation of some mutants compared to wt DHHC2 (^⁎⁎^, p ≤ 0.01; ^⁎⁎⁎^, p ≤ 0.001). The dashed and filled lines represent the upper and lower ratios of endosomal enrichment/depletion that were calculated in Fig. 1. (C) Representative confocal images of PC12 cells co-expressing the wt mCHERRY-zDHHC2 protein (*left panel*) and the two GFP-tagged mutants of zDHHC2 that are significantly enriched in endosomes (*middle panel*). Both are represented in FIRE pseudocolour. The right panel is the merge of the left and middle panels, with wt mCHERRY-zDHHC2 coloured in Magenta and GFP-tagged proteins coloured in Green. Scale bar represents 5 μm. (D) Evidence that the phospho-mimetic mutations affect the activity of the L334/335 endocytic motif but do not affect the activity of the N357_P358 site. The fluorescence intensity values were processed, analysed and presented as described in (B) (n = 9 wt zDHHC2 cells, 12 T340/342A_S344A/345A cells, 10 T340/342E_S344A/345D cells, 11 L334/335A_T340/342E_S344/345D cells and 10 T340/342E_S344/345D_N357A cells). (E) Representative confocal images of PC12 cells are presented as described in (C). (For interpretation of the references to colour in this figure legend, the reader is referred to the web version of this article.)

Finally, we were interested in the reason why the 1–340 mutant shown in Fig. 2 had reduced plasma membrane levels compared with the 1–350 mutant. Indeed, T340 has been identified as a phosphorylation site in human zDHHC2 (S341) and there are several other serine and threonine residues in this region of the protein (T342, S344 and S345). As shown in Fig. 8, phosphomimetic mutation of the threonines or serines in pairs led to a loss of plasma membrane localisation of zDHHC2. These effects required an intact dileucine motif, implying that potential phosphorylation of residues in this region of zDHHC2 (340–350) also has the potential to regulate the efficacy of zDHHC2 endocytic signals, and offering an explanation for the localisation of the 1–340 mutant shown in Fig. 2.

### 3.5 Mutation of specific serine and threonine residues modulates zDHHC2 localisation in primary neurons

**Fig. 9:**
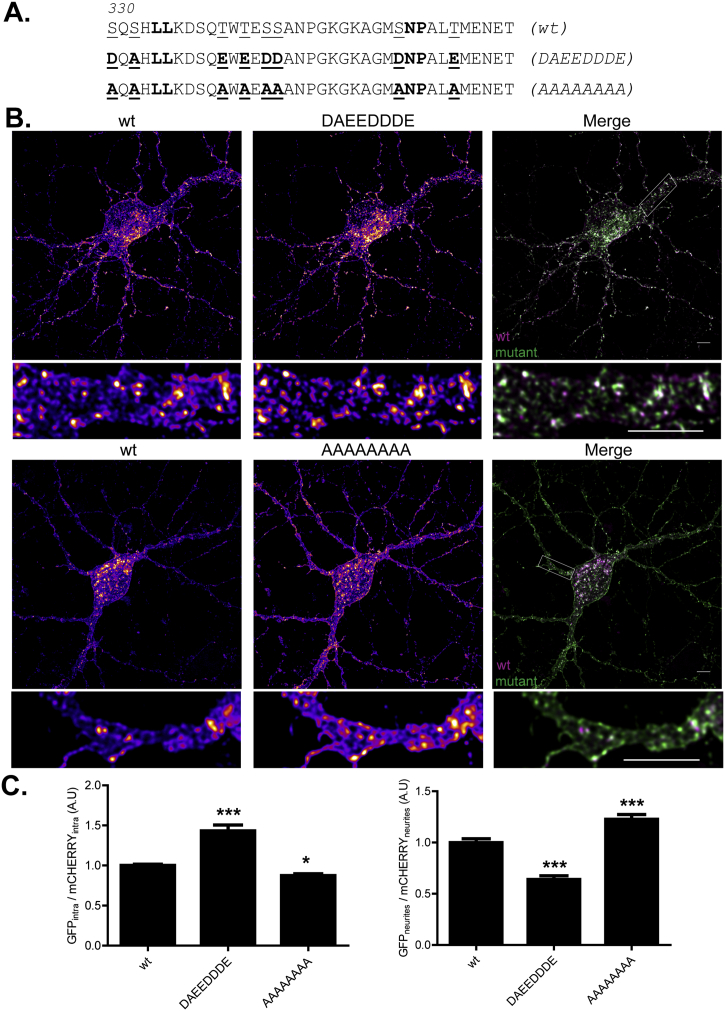
Phospho-mimetic and phospho-null mutations affect the localisation of zDHHC2 in primary rat hippocampal neurons. (A) Amino acid sequence of the 330–366 region of zDHHC2. The serine and threonine residues that might potentially modulate zDHHC2 localisation are underlined. All the phospho-mimetic and phospho-null mutations were combined to give rise to 2 new mutants, named DAEEDDDE and AAAAAAAA. These GFP-tagged mutants were expressed together with the wt mCHERRY-zDHHC2 protein in primary rat hippocampal neurons. (B) Representative confocal images of 13 DIV primary rat hippocampal neurons cells co-expressing wt mCHERRY-zDHHC2 (*left panel*) and the GFP-tagged mutants of zDHHC2 (*middle panel*). Both are represented in FIRE pseudocolour. Below each cell is a zoom image of a neurite region (defined by a box in the corresponding full size merged image).The right panel is the merge of the left and middle panels, with wt mCHERRY-zDHHC2 coloured in Magenta and GFP-tagged proteins coloured in Green. Scale bar represents 5 μm. (C) Quantification of the intracellular depletion or enrichment of zDHHC2 mutants and of their neurite enrichment or depletion compared to the wt protein. *Left panel*. The fluorescence intensity values of the intracellular membrane compartment (intra) as a fraction of the whole soma fluorescence were calculated as described in material and methods for both GFP and mCHERRY. The graph shows the normalized mean ratio ± SEM of endosomal fluorescence of the GFP construct relative to wt mCHERRY-zDHHC2. *Right panel*. The mean fluorescence intensity of the neurites related to the soma was calculated for both GFP- and mCHERRY-tagged proteins. The graph shows the normalized ratio ± SEM of neurite fluorescence intensity for the GFP construct relative to wt mCHERRY-zDHHC2 (n = 17 wt zDHHC2 cells, 15 DAEEDDDE and 22 AAAAAAAA cells). Statistical analysis (ANOVA) shows a significant difference in the localisation of the mutants compared to wt zDHHC2 (^⁎^, p ≤ 0.05; ^⁎⁎⁎^, p ≤ 0.001). (For interpretation of the references to colour in this figure legend, the reader is referred to the web version of this article.)

Finally, we sought to determine if mutations of the identified serine and threonine residues in zDHHC2 had a similar impact on the localisation of the enzyme in hippocampal neurons. Thus, we constructed two mutant proteins, where all serine/threonine residues were mutated to alanine or aspartate/glutamate (Fig. 9A). The phospho-mimetic substitutions had the same effect as in PC12 cells: an increase in endosome levels (Fig. 9B & C). In contrast, the alanine substitutions enhanced plasma membrane localisation (Fig. 9B & C). Furthermore, when the presence of zDHHC2 in neurites was quantified as before, there was a decreased presence (relative to cell body staining) of the aspartate/glutamate mutant and an increased presence of the alanine mutant in neurites.

## 4 Discussion

We have previously shown that zDHHC2 cycles between the plasma membrane and endosomes, and that this was regulated by C-terminal sequences (Greaves et al., 2011). We now identify 2 distinct endocytic sequences within this region It is worthwhile noting that zDHHC2 is able to form dimers/oligomers (Lai and Linder, 2013) and thus it is possible that co-expression of wild-type zDHHC2 may facilitate the trafficking of mutant zDHHC2 proteins that have perturbed endocytic signals. Thus, although co-expression of wild-type and mutant zDHHC2 proteins provides a convenient approach to detect changes in the localisation of mutant proteins, it is possible that the presence of wild-type zDHHC2 could limit the magnitude of the measured localisation changes.

**Fig. 10:**
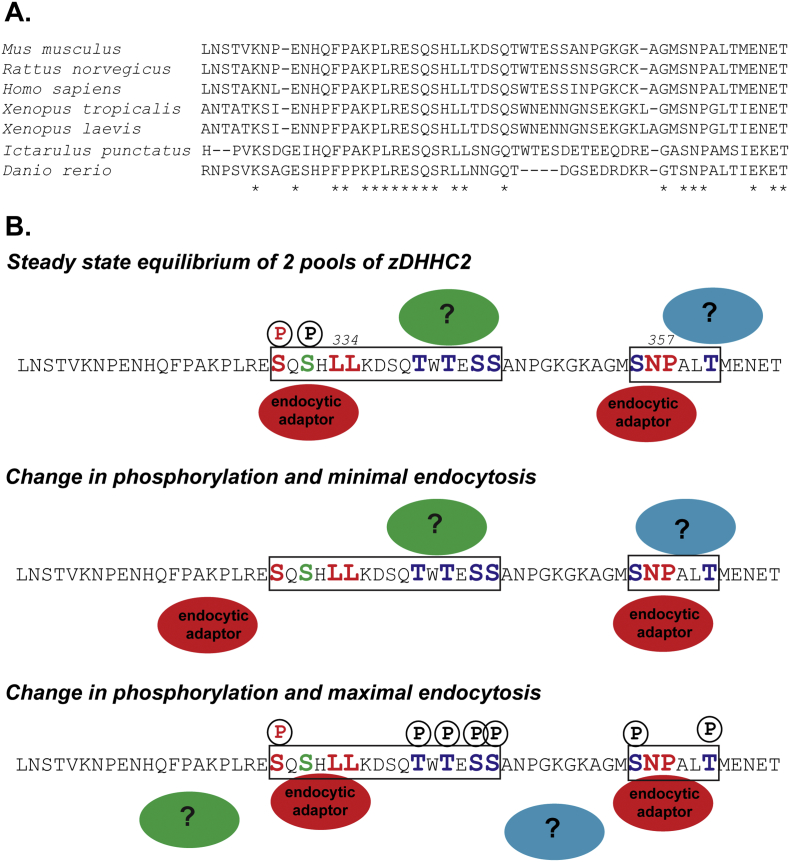
Conservation of the endocytic sequences SQSxxLL and SNP among Vertebrates (A) and Proposed mechanism for the regulation of zDHHC2 localization by phosphorylation (B). (A) A Blast search was performed with *Mus musculus* zDHHC2 protein sequence (NP_848482) against all the reference sequences present in the NCBI database. Among them only the curated sequences were chosen here and aligned with the COBALT program. Those sequences are NP_659564.2 (*Rattus norvegicus*), NP_057437.1 (*Homo sapiens*), NP_001072234.1 (*Xenopus* (*Silurana*) *tropicalis*), NP_001187817.1 (*Ictalurus punctatus*) and NP_001013510.1 (*Danio rerio*). The sequences shown start at amino acid 310 and end at amino acid 366 of the mouse protein. The asterisks highlight residues that are 100% conserved between the 7 sequences. (B) The diagram shows the C-terminal 57 amino acids of mouse zDHHC2, with the two identified endocytic sites (framed by the black boxes) and the core amino acids in Red (including phospo-Ser 330). A possible mechanism of action of the two identified endocytic sites is the following: at basal state there would be a competition between endocytic adaptors (Red circles) and unidentified protein(s) (Blue and Green circles) that would stabilize a pool of zDHHC2 at the plasma membrane. A simpler alternative would be that the affinity between zDHHC2 and endocytic adaptors is not optimum when the surrounding serines and threonines (Blue colour) are not phosphorylated (except for S332, Green colour), thereby allowing for the stabilization of a pool at the plasma membrane. Dephosphorylation of S330 would inactivate the dileucine motif and shift the balance toward the plasma membrane. Conversely, increased phosphorylation around the endocytic sites would either reduce the affinity of zDHHC2 with these unidentified plasma membrane proteins or increase its affinity for endocytic adaptors, thereby increasing its endocytosis and shifting the balance toward endocytosis. Another alternative is that phosphorylation mediates conformational changes within zDHHC2 C-terminal tail and unmasks the endocytic sites. (For interpretation of the references to colour in this figure legend, the reader is referred to the web version of this article.)

Fig. 10B summarizes our findings and proposes a possible mechanism regulating the activity of the endocytic sequences by phosphorylation. Interestingly, both sequences, SxxxLL and NP deviate from the canonical endocytic D/ExxxLL and (Fx)NPxY/F signals. Interestingly, an alignment of the 95 zDHHC2 mammalian reference sequences that can be found in the RefSeq NCBI database highlights the conservation of both the dileucine sequence (in 100% of the sequences) and of the NPALT motif (in 94/95 sequences). Moreover, the alignment of the 7 curated protein sequences found in the NCBI full database shows that the SQSxLL and the SNP motifs are also conserved between Vertebrates, including Mammals, Amphibians, and Bony fishes (Fig. 10A). The amino-acids present in between are less well conserved, suggesting that both endocytic sites play a key role in zDHHC2 function. An alignment of the C-terminal domain of all mouse zDHHCs shows that zDHHC15 and zDHHC20 are the most similar for this region. None of these proteins contains a SNP motif, suggesting that this endocytic site might be particularly important for zDHHC2 function. However, both display a dileucine sequence (SxxxLL) that is not surrounded by phosphorylable amino acids, suggesting that any fine-tuning of the potential endocytic or trafficking activity of the dileucine sequence is specific to zDHHC2.

The SQSHLL motif is implicated in the endocytosis of zDHHC2 since the mutation of the core LL sequence increases the presence of zDHHC2 at the plasma membrane. D/ExxxLL motifs are recognized by the AP2 clathrin adaptor. The negatively charged amino-acid (D/E) at position − 4 of the LL motif occurs on a hydrophilic patch of AP2 with an overall positive charge (Kelly et al., 2008). In accordance with this, we found that substituting Ser − 4 (S330) within zDHHC2 for the acidic amino acid aspartate enables the dileucine sequence to function as an endocytic signal whereas substitution with an alanine reduces its activity and enhances the presence of the protein at the plasma membrane. Asp is commonly used in site-directed mutagenesis to simulate the phosphorylated status of a serine, and these results highlight the potential of phosphorylation to modulate the activity of this dileucine motif in zDHHC2. Very few examples of such a regulation have been found previously. Early studies showed that the phosphorylation of a SxxxLL motif within the granulocyte colony-stimulating factor receptor G-CSF-R was an important determinant in the rate of its ligand-induced internalisation (Aarts et al., 2004). Another example is the downregulation of the Interleukin-6 receptor subunit gp130 via the phosphorylation of Ser − 4 within a SxxxLL motif by the stress kinase MK2 (Radtke et al., 2010). In a third example, neuronal activity have been shown to modulate the cell surface expression of the G-protein-activated inwardly rectifying potassium channels (GIRKs) via the phosphorylation or dephosphorylation of a serine residue (S9) which is part of an endocytic SxxxVL motif (Chung et al., 2009). The activation of NMDARs in pyramidal neurons triggers the dephosphorylation of S9 by Protein Phosphatase 1, and this in turn induces the recycling of GIRKs from endosomes to the plasma membrane of the soma and neurites.

Interestingly, S330 is part of a SQ motif. The phosphorylation of SQ motifs in neurons is bi-directionally regulated by synaptic activity (Siddoway et al., 2014). Blocking synaptic activity with TTX decreases the phosphorylation level of the sequence-specific SQ phosphoproteome below basal levels, whereas inducing synaptic activity and membrane depolarization with high KCl increases the phosphorylation of SQ-containing proteins. The dephosphorylation of S330 within the SQ motif in response to TTX would inactivate the endocytic motif and stabilize zDHHC2 at the plasma membrane whereas its phosphorylation following membrane depolarization would induce zDHHC2 endocytosis via a now functional pSQxxLL. Pharmacological data suggest that the SQ kinases involved are neuronal Ataxia telangexia mutated (ATM) or Ataxia telangexia mutated and Rad3 related (ATR) kinases. Interestingly, S330 has a very strong probability of being a potential substrate of ATM kinase (score of 0.97 determined by KinasePhos (Huang et al., 2005)). In addition, the data we present suggest that the potential phosphorylation of S332, which has already been identified as a phosphorylated residues in zDHHC2, could fine-tune the activity of this sorting motif.

The second internalisation signal identified is also clearly atypical. Indeed, we found that both N357 and P358 were involved in the endocytosis of zDHHC2 as their mutation into alanine increases its plasma membrane expression; N357 appeared to be particularly important. However, the amino acids located upstream and downstream do not match any consensus endocytic sequence such as (F/Y)xNPxY or NPxY. NPxY motifs bind to clathrin adaptor proteins (such as Numb, Dab2, Idol, ARH, X11 or Snx17 (Traub and Bonifacino, 2013, Zhang et al., 1997)) through their phosphotyrosine binding domains (PTB). However, despite their name, PTB domains have been found to be capable of binding an increasing variety of sequences deviating from the consensus NPxY, some that do not even contain any Y at all (Forman-Kay and Pawson, 1999, Zhang et al., 1997, Borg et al., 1996). We hypothesize that the core N357_P358 sequence of zDHHC2 binds to a clathrin adaptor protein. Furthermore, this binding and subsequent endocytosis might be enhanced by a potential phosphorylation of the flanking S and T residues (S356 and T361), as we found that phosphomimetic mutation of these residues led to a loss of plasma membrane localisation via effects on the NP motif.

Synaptic strength is controlled through balanced phosphorylation and dephosphorylation (Woolfrey and Dell'Acqua, 2015). The activity-sensitive phosphorylation and dephosphorylation of residues in zDHHC2 could rapidly switch its localisation between the plasma membrane and the endosomes. zDHHC2 indeed plays crucial roles in both compartments following the induction of chemically induced long-term potentiation (cLTP) or homeostatic upscaling. When localised to recycling endosomes zDHHC2 regulates the exocytosis of the endosomes and AKAP79/150 recruitment to the spines (Woolfrey et al., 2015). After translocation and integration at the spine membrane, zDHHC2 S-acylates and anchors PSD95 to the PSD (Noritake et al., 2009, Fukata et al., 2013). The role of phosphorylation in the activity-sensitive trafficking of another S-acyltransferase has been uncovered recently (Brigidi et al., 2015). zDHHC5 mainly resides at the spine membrane at resting state. zDHHC5 is tyrosine phosphorylated by Fyn within a YxxL motif at basal state, preventing the binding of AP2, hence stabilizing zDHHC5 at the plasma membrane. cLTP activation decreases Fyn activation and zDHHC5 phosphorylation and this triggers the subsequent endocytosis of zDHHC5. These findings together with the present study clearly emphasise the potential role of phosphorylation cycles in the regulation of zDHHC enzymes. The differential effects of serine/threonine and alanine mutations on zDHHC2 localisation are indeed consistent with a role for phosphorylation of some of these sites in regulating the efficacy of the identified internalisation motifs. However, it is also possible that these serine/threonine residues are part of a larger endocytic motif that is recognized by adaptor proteins and that they do not undergo phosphorylation to a significant extent in vivo. Whilst this is a point that must be considered, it would nevertheless be more likely that any amino acid substitutions introduced into an endocytic motif would disrupt protein interactions. Given the differential effects of alanine and aspartate/glutamate mutations, this does not appear to be the case and we favour the view that these substitutions are mimicking the effects of phosphorylated or dephosphorylated states of zDHHC2. Moreover, although we hypothesize that the phosphorylation and dephosphorylation events might directly affect the binding of adaptor proteins to the endocytic sites, other mechanisms can also be evoked to explain a regulation of zDHHC2 endocytosis by phosphorylation (Fig. 10B). Phosphorylation could modulate zDHHC2 conformation or binding to regulatory proteins, thereby masking and unmasking the endocytic sites and/or stabilizing it either at the plasma membrane or within the endosomal compartment. As a summary, whilst the results of the present study clearly show the potential of phosphorylation to regulate the efficacy of sorting signals in zDHHC2, in future work it will be important to examine in more detail the residues that undergo phosphorylation in zDHHC2, whether any phosphorylation events are affected by neuronal activity, and the relevant kinases and phosphatases involved.

Interestingly, we have also found that the same mutations that are affecting the surface versus endosome localisation of zDHHC2 both in PC12 and in primary neuronal cells were also involved in its trafficking to neurites. Transmembrane proteins synthesized in the soma have to travel hundreds of micrometres to reach distal dendrites. Two main models have been proposed: lateral diffusion of proteins within the plasma membrane from the soma, or trafficking of vesicles followed by their subsequent integration at the dendritic membrane (Cognet et al., 2006, Chen et al., 2007). The first model could explain our results in a very simple manner: an increased presence at the plasma membrane of the L334/335A_N357A or the AAAAAAAA mutants would allow for these proteins to diffuse further along the neurites. Conversely, an increased endocytosis of the DAEEDDDE mutant would restrict its location to the soma and its diffusion along the neurites. However, this model cannot account for all the trafficking events observed in neurons and numerous studies are also uncovering a complex web of vesicles trafficking from the soma to neurites (Valenzuela and Perez, 2015, Bonifacino, 2014). Distinct vesicles are thought to direct the different trafficking of AMPAR from NMDAR, for example (Jeyifous et al., 2009). Whereas AMPARs are trafficked to the plasma membrane dendrites via the conventional somatic Golgi network, NMDARs are diverted to a specialized endoplasmic reticulum subcompartment that merges with dendritic Golgi outposts (Jeyifous et al., 2009). Different motor proteins would be involved in the trafficking of distinct vesicles along the neurites. Additionally, the differentiation between proximal and distal dendrite localisation also seems to involve a sorting into different vesicles at the Golgi apparatus. Indeed, voltage gated potassium channels (Kv channels) Kv2.1 and Kv4.2 have been shown to be sorted into distinct vesicles at the Golgi apparatus, explaining their proximal and distal, respectively, localisation within dendrites (Jensen et al., 2014). Kv4.2 vesicles had an increased velocity along dendrites compared to Kv2.1 containing vesicles, and associated with particular motor proteins and cytoskeletal elements. The peptide sequences that are dictating a differential sorting between these distinct populations of vesicles are still largely unknown. Early studies have found that signals mediating the dendritic targeting of the transferrin receptor (TfR) might overlap with endocytic signals (West et al., 1997), and there is a correlation between the enhanced plasma membrane association of some TfR mutants and their disrupted dendritic targeting. Similarly, the mutation of a dileucine sequence within Kv4.2 disrupts its dendritic targeting (Jensen et al., 2014, Rivera et al., 2003). Since endocytic sequences such as D/ExxxLL can recruit diverse endocytic adaptors, such as AP1 at the Golgi membrane for example, it would therefore be logical that a certain amount of overlap exists between plasma membrane endocytosis, sorting at the Golgi apparatus and polarized trafficking (Bonifacino, 2014). In summary, our own results suggest that peptide sequences mediating the subcellular localisation of zDHHC2 in neurons are present in its C-terminal cytoplasmic part. The mutations that were introduced within the C-terminal tail of zDHHC2 might modify its sorting at the level of the Golgi apparatus and this was uncovered when expressed in polarized cells such as neurons. Finally, our results suggest an overlap between the sequences dictating neurite trafficking and endocytic signals.

## Acknowledgements

We are grateful to Gillian Robb of the Centre for Biophotonics, University of Strathclyde for assistance with confocal microscopy.

This work was funded by BBSRC grants BB/J006432/1 and BB/L022087/1.

## Author contributions statement

CS, designed and performed all experiments, analysed the data and wrote the manuscript; LR, performed the experiments; JG, generated plasmid constructs for analysis, TJB, designed the experiments and wrote the paper; LHC, designed the experiments and wrote the paper.

## Competing financial interests

None.
